# Intra-Arterial Glycoprotein IIb/IIIa Inhibitor Treatment for Symptomatic Intracranial Atherosclerotic Stenosis Presenting as Large Vessel Occlusions

**DOI:** 10.7759/cureus.9243

**Published:** 2020-07-17

**Authors:** Aditya Srivatsan, Visish M Srinivasan, Stephen Chen, Peter Kan, Jeremiah N Johnson

**Affiliations:** 1 Neurosurgery, Baylor College of Medicine, Houston, USA; 2 Interventional Radiology, MD Anderson Cancer Center, Houston, USA

**Keywords:** ischemic stroke, thrombectomy, intracranial atherosclerosis, gp iib/iiia inhibitors

## Abstract

Introduction

There is no consensus on the optimal treatment for acute ischemic stroke (AIS) large vessel occlusions (LVOs) or near-occlusions with underlying intracranial atherosclerotic stenosis (ICAS). We report the first American series using intra-arterial (IA) glycoprotein IIb/IIIa inhibitors (GPIs) as a stand-alone revascularization technique for ICAS presenting with large vessel ischemic syndromes.

Methods

Records at two centers of 140 patients presenting with AIS undergoing stroke intervention from January 2017 to June 2019 were retrospectively reviewed. Patients treated with IA GPIs were identified, and baseline factors, imaging, procedural characteristics, hospital course, and outcomes were collected. Six patients with ICAS underlying their acute symptomatic near occlusion or LVO were treated with IA GPI. Four near-occlusions were treated with IA GPI as the first-line therapy, while two LVOs were treated with IA GPI as an adjunct therapy to thrombectomy.

Results

The mean age was 61.3 years (range 36-79), presentation National Institute of Health Stroke Scale (NIHSS) was 10 (4-18), time from last seen well to treatment was 434.5 minutes (164-1290), and time from groin puncture to revascularization was 67.3 minutes (26-94). Three patients received intravenous (IV) tissue plasminogen activator (tPA), and all patients received an IA weight-based GPI infusion. Five patients had thrombolysis in cerebral ischemia (TICI) 3, and one patient had TICI 2b. The mean discharge NIHSS was 2.5 (0-8). The mean modified Rankin scale was 1.3 (range 0-4) at discharge and .8 at three months. No patients had a postprocedural symptomatic hemorrhage.

Conclusion

Our results highlight the utility of IA GPI administration as the first-line therapy for symptomatic ICAS near occlusions or as a rescue technique after failed thrombectomy for LVO patients suspected of underlying ICAS.

## Introduction

Five landmark randomized controlled trials (RCTs) in 2015 established mechanical thrombectomy as the treatment of choice for acute ischemic stroke (AIS) due to anterior circulation large vessel occlusion (LVO), and the recent DEFUSE 3 (Endovascular Therapy Following Imaging Evaluation for Ischemic Stroke) and DAWN (DWI or CTP Assessment with Clinical Mismatch in the Triage of Wake-Up and Late Presenting Strokes Undergoing Neurointervention with Trevo) trials have even expanded the thrombectomy window in certain AIS cases [1-7]. The HERMES (Highly Effective Reperfusion evaluated in Multiple Endovascular Stroke) meta-analysis of individual data from the five RCTs found that recanalization failed in ≈29% of patients who underwent mechanical thrombectomy, and a major cause for this was underlying intracranial atherosclerotic stenosis (ICAS), as thrombectomy devices are primarily designed for embolus retrieval [8]. Thrombo-occlusion secondary to ruptured plaque in the presence of ICAS is a unique cause of LVO due to its propensity for unsuccessful stent retrieval passes and the potential that repeated mechanical revascularization attempts may worsen the already damaged plaque endothelium, resulting in rapid platelet activation and vessel re-occlusion [9]. Thus, arterial occlusion related to symptomatic target vessel ICAS has been associated with longer procedure times and poorer clinical outcomes [10]. Though ICAS is particularly prominent in Asian countries, showing rates of 15% and 35% for patients with anterior circulation occlusion and vertebrobasilar occlusion respectively, a French study reported intracranial atherosclerotic stenosis rates of 5.5% for patients who underwent endovascular revascularization treatment [11-12]. Furthermore, a recent United States study showed that in 1765 patients between the ages of 67 and 90 years, ICAS was prevalent in 31% of participants and 9% had ICAS ≥50% [13]. Thus, it is imperative that practitioners are proficient in recognizing and treating cases of LVO due to ICAS.

Intra-arterial (IA) glycoprotein IIb/IIIa inhibitors (GPI), such as eptifibatide and tirofiban, are drugs that inhibit the GpIIb/IIIa receptor on the surface of platelets, thus preventing platelet aggregation and thrombus formation. Though commonly used for percutaneous coronary intervention, two groups recently attempted GPI use as a rescue remedy for ICAS patients after failed thrombectomy [14-15]. In this study, we report the first American experience using intra-arterial (IA) glycoprotein IIb/IIIa inhibitors (GPI) to achieve target vessel recanalization in patients undergoing acute stroke intervention procedures who were suspected intra-procedurally to have underlying ICAS. Resolving the acute flow-limiting stenosis and avoiding an intracranial stent placement in LVO patients may be advantageous, as it allows greater flexibility for halting GPIs with less concern for subsequent long-term segment stent thrombosis if a postprocedure hemorrhagic conversion is encountered. If successful revascularization is achieved, this strategy avoids well-known complications of intracranial stenting in the setting of ICAS occluding nearby eloquent perforator vessels through compressed plaque and thrombus. In addition, we review our indications for GPI administration, including the radiographic and angiographic pre- and intra-procedural indicators of underlying ICAS.

## Materials and methods

Patients

Records of 140 patients presenting with AIS who underwent acute stroke intervention at two comprehensive stroke centers between January 2017 and June 2019 were retrospectively reviewed. Symptomatic patients presenting to our emergency department as stroke alerts with National Institute of Health Stroke Scale (NIHSS) scores ≥ 4 and computed tomography angiogram (CTA) demonstrating that either high-grade large vessel intracranial stenosis (≥ 70% stenosis defined by the WASID (Warfarin-Aspirin Symptomatic Intracranial Disease) criteria) or large vessel occlusions were analyzed. A retrospective analysis was performed on patient data that was prospectively collected (H-33379) [16]. This study was approved by the institutional ethics committee at both centers, and informed consent for endovascular therapy was obtained from a family member for all patients.

Endovascular Therapy

Inclusion criteria for endovascular therapy were the following: presentation within six hours of stroke onset for anterior circulation stroke; baseline NIHSS ≥ 4 with no hemorrhage or large territory infarct detected on cranial CT; occlusion or near occlusion of the internal carotid artery (ICA) or middle cerebral artery (MCA) artery detected with CTA and confirmed via catheter angiography; baseline modified Rankin Scale (mRS) score ≤ 2. Patients with NIHSS below 6 were taken to intervention based on CTA and CT perfusion imaging confirming LVO or near-occlusion with corresponding large perfusion deficit in the setting of debilitating clinical symptoms such as aphasia or waxing and waning symptoms considered harbingers of early neurological decline.

The endovascular stroke intervention patients in which IA GPIs were administered for suspected ICAS underlying symptomatic high-grade stenosis or occlusion were identified and included in this series. We also outlined the clinical and radiographic factors we used to diagnose ICAS and rule out other similar diseases. In this study, IA GPI infusion was initiated as primary therapy for patients who were determined to have high-grade stenosis with downstream flow restriction. IA GPIs were administered as an adjunct or bail-out strategy in patients who underwent mechanical thrombectomy with a stent-retriever during which radiographic cues or lesion behavior led the operator to suspect underlying ICAS (i.e. immediate post-stent-retriever pass reperfusion with residual stenosis radiographically resembling underlying ICAS, followed by progressive vessel re-occlusion over several minutes).

The GPIs tirofiban or eptifibatide were used in this series with the IA dose calculated using acute coronary syndrome (ACS) intravenous (IV) dose recommendations from the manufacturer. Treatment typically involved 0.5 mg of GPI diluted with 8 mL of normal saline injected with an infusion rate of 1 ml/min through a catheter in the internal carotid artery proximal to the stenosis. Total GPI dosage ranged from .5-1.0 mg. Immediately after infusion and 10 minutes after completion of infusion, follow-up angiography was performed. Local anesthesia was used in all patients for cerebral angiography and endovascular therapy.

Outcome Measures

Needle puncture of the common femoral artery defined the start time of endovascular therapy. The interval between the time of onset of symptoms and the start of endovascular therapy demarcated the time to procedure. P2Y12 reactivity unit (PRU) testing is recommended at 24 hours post-oral loading of aspirin (ASA)/Plavix to identify Plavix non-responders. All patients underwent NCCT six to 24 hours after endovascular therapy. Symptomatic intracranial hemorrhage was defined as any intracranial hemorrhage that caused neurological deterioration and a ≥ 4-point increase in the NIHSS score. The final angiogram was used to assess reperfusion status according to the modified treatment in cerebral infarction (m-TICI) scale, and successful reperfusion was considered an m-TICI grade of 2b or 3 [17]. A stroke neurologist evaluated patients’ neurological function within 24 hours of treatment. Three months after treatment, the functional outcome was assessed by a stroke neurologist or endovascular neurosurgeon using the mRS score. The assessment was made through telephone if the patient was unable to visit the outpatient department. An mRS score of ≤ 2 was considered a successful outcome.

## Results

Of the 140 AIS interventions, six patients were determined to have large vessel ischemia due to the underlying ICAS and met the inclusion criteria. Three patients were male and three were female, and the median age was 61.3 years (36-79 years). All six patients had MCA ischemic stroke syndromes with mean presentation NIHSS of 10 (range 4-18). The mean time from last-seen-well to treatment was 434.5 minutes (range 164-1290), and time from groin puncture to full revascularization was 67.3 minutes (range 26-94). Four patients were treated with IA GPI as the first-line therapy without mechanical thrombectomy, while two were treated with IA GPI as a rescue therapy after failed thrombectomy. All six patients had a history of hypertension, two had diabetes mellitus, and one instance of coronary artery disease, seizures, bipolar disorder/depression, thyroid disease, and prior transient ischemic attack (TIA) each were seen. Three of the six patients received IV tissue plasminogen activator (tPA) prior to the intervention. All patients received weight-based IA GPI infusion followed by maintenance GPI drip and subsequent subacute transition to oral antiplatelet therapy. There were no intra-procedural complications. Five patients had TICI 3, and one patient had TICI 2b. There were no post-procedural symptomatic hemorrhages. One patient experienced a self-limited retroperitoneal hematoma that was managed conservatively. The mRS was 1.3 (range 0-4) at discharge, and the mean discharge NIHSS was 2.5 (0-8). The three-month follow-up mRS was available for five out of the six patients, and the mean three-month mRS was .8 for the five available scores. These results are detailed in Table 1.

**Table 1:** Demographic, treatment, and clinical outcome characteristics of our six patients IV rt-PA, intravenous recombinant tissue plasminogen activator; GPI, glycoprotein IIb/IIIa inhibitor; LTSW, last-seen-well; Revasc, revascularization; NIHSS, National Institute of Health Stroke Scale; m-TICI, modified thrombolysis in cerebral infarction score; mRS, modified Rankin Scale; N/A, not available

No.	Age (yrs)	Gender	IV rt-PA	Thrombectomy attempted?	Type of GPI	Site of Occlusion	LTSW to Puncture Time (min)	Groin Puncture to Revasc Time (min)	Pre-intervention NIHSS	NIHSS at Discharge	m-TICI	mRS at Discharge	mRS at 3 Months
1	63	M	Y	N	Eptifibatide	R-M1	164	90	18	2	3	1	0
2	74	F	N	N	Tirofiban	R-distal M1 & proximal M2	205	94	5	0	3	0	0
3	65	F	Y	N	Tirofiban	L-proximal inferior division of M2	331	56	11	0	3	0	0
4	51	M	Y	Y	Eptifibatide	L-M1	371	86	12	5	3	3	N/A
5	79	F	N	Y	Tirofiban	R-M2 inferior division	246	26	10	8	2b	4	4
6	36	M	N	N	Tirofiban	L-M1	1290	52	4	0	3	0	0

Illustrative cases

Case 1

**Figure 1:**
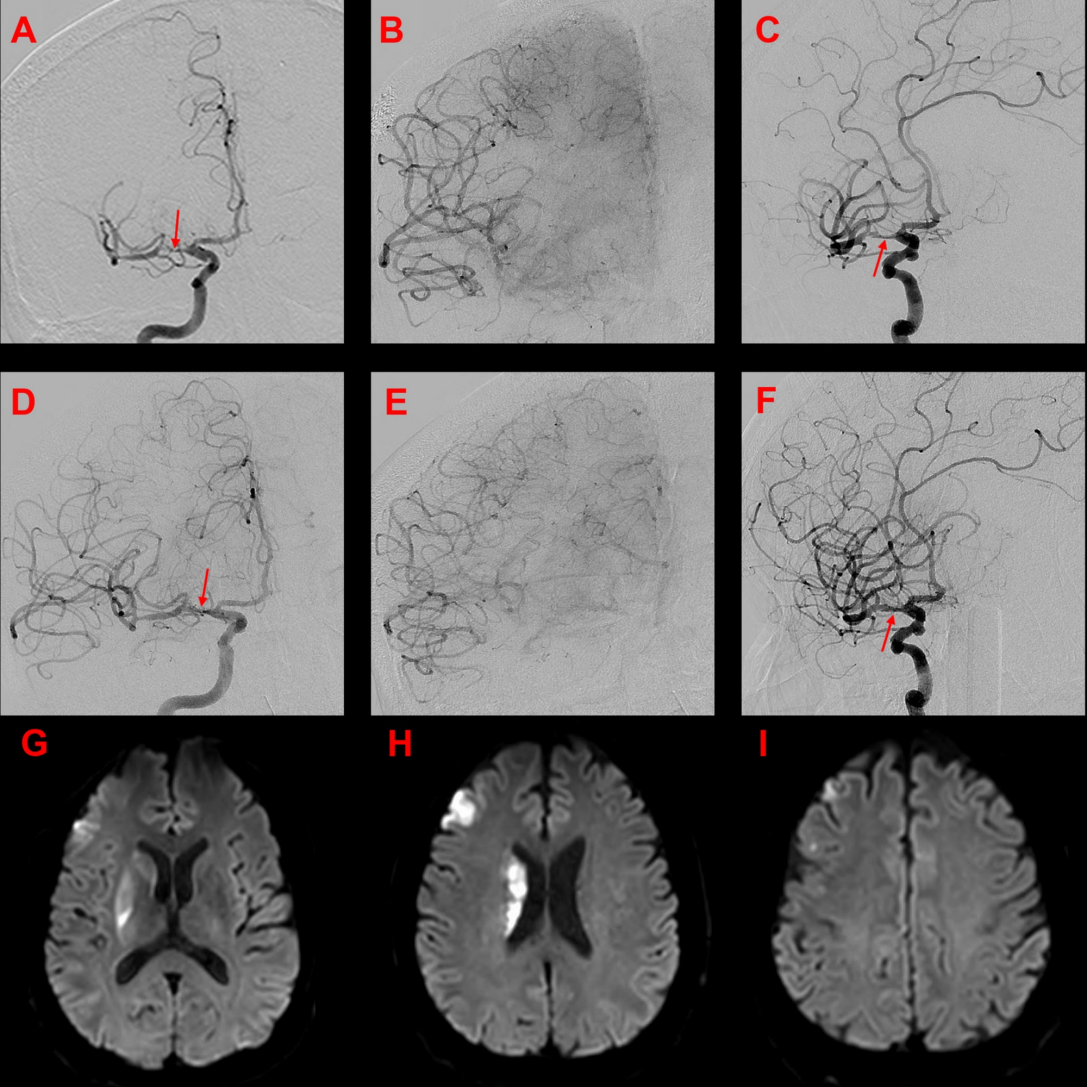
Angiogram of case 1 Pre-intervention AP (A) early arterial, (B) capillary phase images, and (C) AP magnified oblique demonstrating high-grade right MCA stenosis (arrow), associated thrombus, and MCA arterial delay. Post-intra-arterial eptifibatide infusion (D) early arterial, (E) capillary phase images, and (F) AP magnified oblique demonstrating a notable reduction in M1 stenosis (arrow), reduction in thrombus burden, and increased MCA territory perfusion with lessened delay. Post-procedure MRI (G-I) axial DWI sequence shows a limited M1 perforator territory infarct and modest scattered embolic strokes. AP: anteroposterior; MCA: middle cerebral artery; MRI: magnetic resonance imaging; DWI: diffusion-weighted imaging

A 63-year-old, right-handed Hispanic male was flown to the emerging department (ED) experiencing acute -onset left hemianesthesia, dense left hemiplegia, left facial paresis, dizziness, and dysarthria for one hour with an NIHSS of 18. He had no recorded prior medical history and had not seen a physician in many years. Baseline noncontrast head CT (NCCT) was negative for hemorrhage, and IV tPA was administered. CTA showed proximal right M1 occlusion, and the patient was taken for thrombectomy. During the procedure, angiographic features suggested that the patient had right M1 high-grade stenosis, underlying atherosclerosis, and superimposed thrombus resulting in near-complete MCA perfusion delay (Figure 1). IA tPA was infused with no improvement, so a calculated weight-based cardiac dose of eptifibatide totaling 7 mg was infused over 10 minutes. Angiographic images showed significant improvement in the degree of stenosis and perfusion of the downstream MCA territory. Therefore, no stent was placed, and the patient was maintained on a continuous weight-based IA eptifibatide infusion (75 mg in 100 mL) at a rate of 11.6 mL/hour and a dosage of 2 mcg/kg/min for 24 hours.

The patient’s exam slowly improved, and by 18 hours after initial eptifibatide administration, the NIHSS was three, and the patient was walking without assistance. Magnetic resonance imaging (MRI) 24 hours after tPA administration showed scattered, multifocal, small volume infarcts in the right MCA territory. At 20 hours post-procedure, the patient was loaded with aspirin 650 mg and Plavix 450 mg by mouth, and the eptifibatide drip was discontinued two hours later. Within 30 minutes of the discontinuation of the eptifibatide drip, the patient worsened neurologically, lost the ability to lift his left arm against gravity, and experienced a recurrence of facial droop and dysarthria. The eptifibatide drip was thus restarted, and he recovered his neurological function once again to an NIHSS of three.

The eptifibatide was subsequently maintained for four additional hours and weaned off. The patient remained neurologically stable and was discharged home post-procedure Day 5 with an NIHSS score of two and an mRS of one with instructions to continue aspirin 325 mg and Plavix 75 mg daily for a minimum of 90 days and at the discretion of the treating neurologist beyond that (in accordance with the 2011 SAMMPRIS (Stenting and Aggressive Medical Management for Preventing Recurrent Stroke in Intracranial Stenosis) trial) [18]. At the one-year follow-up, the patient has recovered neurologically, is an mRS zero, has not experienced any recurrent ischemic symptoms, and remains on dual antiplatelet therapy.

Case 2

A 51-year-old Caucasian male presented to the ED two hours after last-known-well with an NIHSS of 12, hemiplegia, and aphasia. The patient had a history of bipolar disorder, depression, and hypertension, but no history of stroke. NCCT was performed immediately, and no intracranial hemorrhage was seen, but a limited left MCA infarct was noted by radiology. IV tPA was administered, and a CTA showed a left M1 segment occlusion. The patient was taken for stroke intervention.

**Figure 2:**
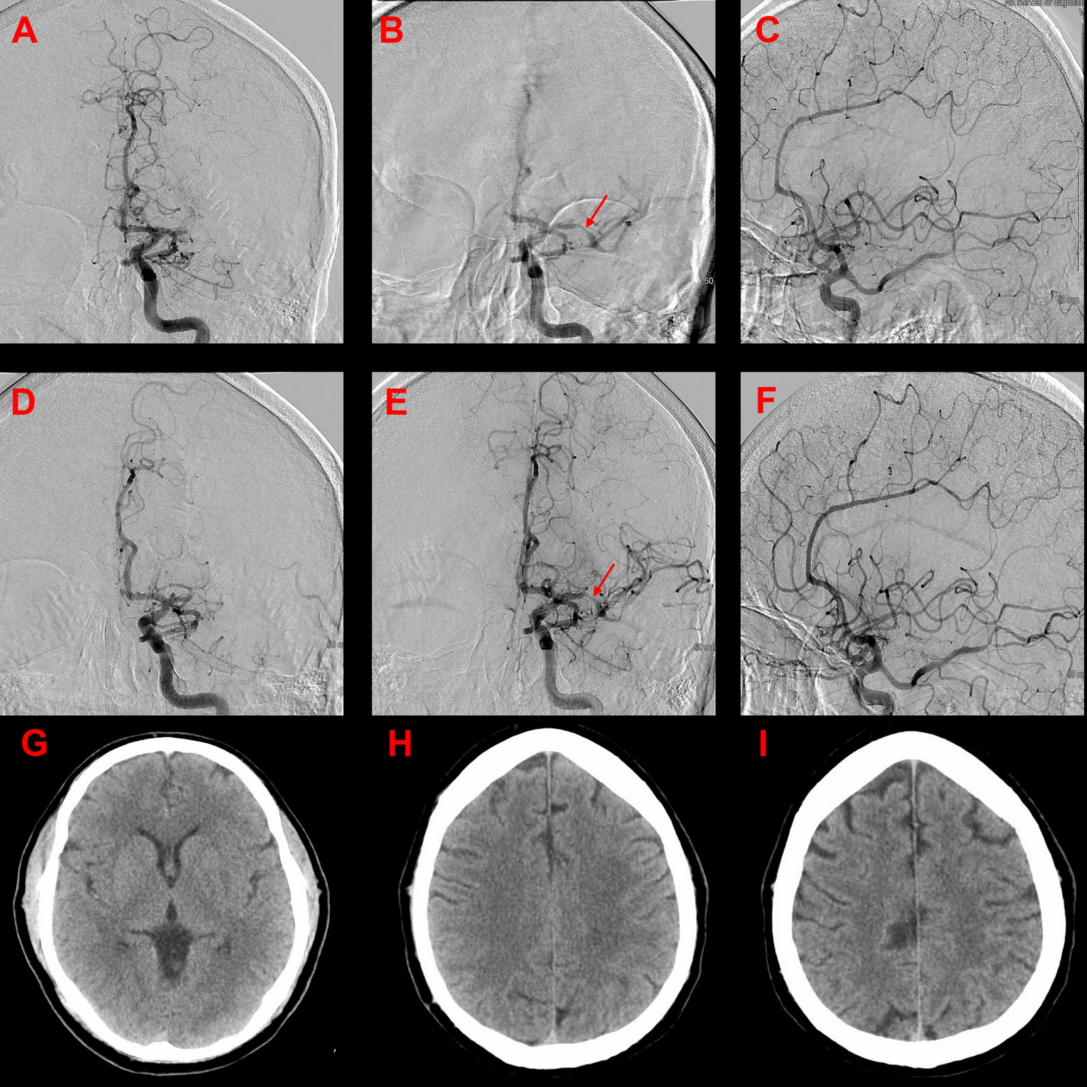
Angiogram of case 2 Initial pre-intervention AP (A) image demonstrating a complete left M1 segment occlusion. Post-stent retriever treatment (B, C), there is restored antegrade flow, but stenosis (arrow) is noted on the AP image. Ten minutes post-thrombectomy, M1 was re-occluded (D). After intra-arterial eptifibatide infusion (E, F), there is restored antegrade flow and reduced stenosis (arrow). CT brain performed at 24 hours (G, I) demonstrates ischemic changes in the basal ganglia and corona radiata. AP: anteroposterior; CT: computed tomography

Mechanical thrombectomy was performed with a stent retriever and successfully opened the left MCA on the first pass (Figure 2). However, there was residual distal MCA stenosis noted, and after 10 minutes of observation, the left MCA reoccluded. IA infusion of a weight-based ACS dose of eptifibatide was then used, and the vessel progressively re-opened with some residual stenosis but good antegrade filling of the MCA territory in phase with the anterior cerebral artery. The patient was maintained on an IV eptifibatide drip and transitioned to oral antiplatelets after post-procedure Day 1 CT brain demonstrated no hemorrhage. However, 48 hours post-procedure, he was noted to have a retroperitoneal hematoma, and the antiplatelets were held until stable hemoglobin levels were established. The patient did well post-procedure, was oriented x3, followed commands, was able to speak with moderate residual expressive aphasia, swallowed independently, and was able to stand and take steps with assistance. The patient was improving and was discharged home with home health after 10 days in the hospital at an NIHSS of five and an mRS of three.

## Discussion

**Figure 3:**
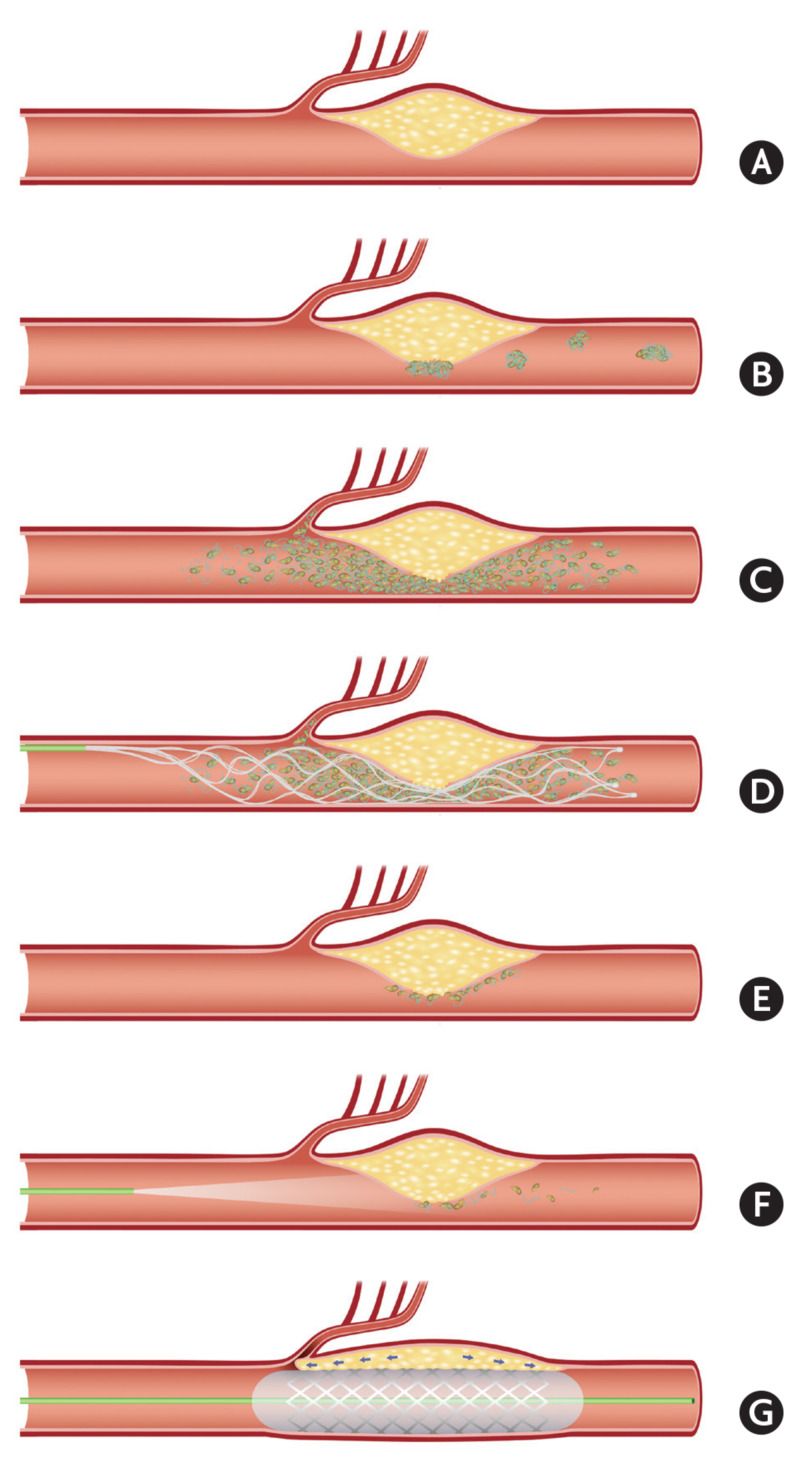
Pathogenesis of intracranial atherosclerotic stenosis Illustrations of ICAS in terms of stroke pathomechanism and endovascular revascularization strategy. (A-C) Pathomechanism of cerebral infarction on ICAS occlusion. Border-zone and scattered infarctions can occur from some microemboli, from in situ thrombosis in the ICAS lesion. Perforator infarctions can also occur from the propagation of the thrombosis. (D) Stent retrieval for ICAS occlusion. Routine first-line thrombectomy can effectively eliminate the major portion of in situ thrombi. (E-F) Endothelial cells are still inflamed and may cause reocclusion. Glycoprotein IIb/IIIa inhibitor can stabilize the irritable endothelium. (G) The location of nearby important perforators should be cautiously evaluated when angioplasty and/or stenting are considered. This procedure can block the perforators, thereby aggravating neurological deficits. *This figure was originally published by Lee et al. and is used here with appropriate copyright approval [19]. ICAS: intracranial atherosclerotic stenosis

ICAS results from the buildup of cholesterol-laden plaque in the walls of cerebral arteries. Over time, plaque buildup can lead to significant arterial lumen narrowing and even vessel occlusion (Figure 3) [19]. Intracranial atherosclerotic plaques typically lie quiescent for a long period of time and may never become symptomatic. However, the overlying endothelial lining can unexpectedly rupture and expose the underlying plaque to blood flow, thus initiating the clotting cascade and subsequent local thrombus formation. Clot accumulation on the plaque can result in the rapid development of flow-limiting luminal stenosis or complete parent vessel occlusion or the clot may accumulate and subsequently dislodge, leading to downstream embolic infarcts.

In patients presenting acutely with suspected LVOs who are subsequently found to have large vessel occlusion or high-grade stenosis secondary to ICAS, acute intracranial stenting has been used to treat suspected ICAS as a bailout method. However, this strategy exposes the patient to the procedural risks of intracranial stenting, including the occlusion of nearby perforators due to the compressing clot and atherosclerotic plaque against the vessel wall. Further, acute stenting in the setting of LVO commits the patient to aggressive antiplatelet therapy while reducing the ability to stop the antiplatelet agents if hemorrhagic conversion is encountered due to potential stent thrombosis. Given the mechanism of symptomatic acute clot overlying ruptured ICAS plaques in this subset of LVO patients, we investigated the administration of IA GPIs in the acute setting to resolve the thrombus overlying the symptomatic intracranial plaque and return the parent vessel to its pre-plaque rupture degree of asymptomatic stenosis. This strategy of reversing the acute platelet-rich thrombus formation, restoring baseline flow, and stabilizing the irritable endothelium may allow neuro-interventionalists to avoid intracranial stent placement and its associated risks in the acute setting [19]. Intra-arterial GPI infusion in the setting of LVO stroke syndrome patients who often have received IV tPA prior to intervention raises the question of the safety of this approach with respect to increased intracerebral hemorrhage (ICH) risk. Kang et al. and Zhou et al. in addition to our data report symptomatic ICH complication rates of 0.7% and 0%, respectively, after GPI use in the setting of LVO secondary to ICAS [14-15]. A similar strategy of acute stenting in the setting of LVO occurs with tandem cervical carotid occlusions and intracranial occlusions [20]. These studies have demonstrated an acceptable peri-procedural symptomatic ICH rate of 5% [20]. Additionally, LVO patients with underlying ICAS who are not treated with GPIs are at high risk for re-thrombosis of the vessels, and those treated with acute intracranial stenting are also subjected to post-procedure ICH risk due to the need for acute GPIs. These patients have an even greater propensity for parent vessel occlusion if antiplatelet agents are stopped or reversed due to ICH development. Hence, we assert that in the setting described herein, stand-alone GPIs resulting in vessel patency and restoration of physiologic antegrade flow is a viable strategy to balance the risk of vessel re-occlusion in patients managed without GPIs vs. risks of acute intracranial stenting.

One of the biggest challenges with ICAS in the setting of LVO syndrome is identifying an LVO secondary to the underlying ruptured atherosclerotic plaque with superimposed platelet aggregation from the more common thromboembolic occlusion based on initial angiographic images. This uncertainty can make initial IA revascularization strategy selection during endovascular procedures challenging. In our experience, several clinical and imaging factors can help differentiate patients with underlying ICAS from those with an embolic occlusion. Clinically, intermittent symptoms attributable to the same vascular territory for hours or weeks before onset, lower than expected NIHSS as compared to the territory occluded, and the fluctuating severity of symptoms can be indicative of underlying ICAS. Regarding non-invasive imaging, focal vessel calcification at the occlusion site on CTA, a halo sign adjacent to high-grade stenosis on CTA, scattered watershed territory infarct on DWI despite occlusion, and unusually robust leptomeningeal collaterals can be indicative of ICAS as well. A recent study also showed that a benign perfusion CT (CTP) profile (Tmax > 4 s/Tmax > 6 s ratio ≥2) was independently associated with underlying ICAS in LVO [21]. During an interventional procedure, high-grade stenosis in the affected vascular territory, the appearance of a focal “bite” out of the target vessel lumen, and initial successful revascularization after thrombectomy pass followed soon thereafter by artery re-occlusion are indicators of ICAS (Table 2).

**Table 2:** Clinical and radiographic factors for detecting intracranial atherosclerotic stenosis NIHSS, National Institute of Health Stroke Scale; CTA, computed tomography angiography; DWI, diffusion-weighted magnetic resonance imaging; Tmax > 4 s, time to maximum of residue function > 4 seconds; Tmax > 6 s, time to maximum of residue function > 6 seconds; LVO, large vessel occlusion

Clinical:
Intermittent symptoms for hours or weeks before onset
Lower than expected NIHSS compared to territory occluded
Fluctuating severity of symptoms
Non-invasive imaging:
Focal vessel calcification at occlusion site on CTA
Halo sign adjacent to high-grade stenosis on CTA
Scattered watershed territory infarct on DWI despite occlusion
Unusually robust leptomeningeal collaterals
Benign CT perfusion profile (Tmax > 4 s/Tmax > 6 s ratio ≥2)
Invasive imaging:
High-grade stenosis in the affected vascular territory with LVO syndrome
Appearance of focal “bite” out of target vessel lumen
Initial revascularization after thrombectomy followed by re-occlusion

Our six patients treated with IA GPIs compared favorably to two similar cohorts from previous studies (Table 3). The first of these two similar studies is a seven-patient cohort from a 2017 report by Zhao et al. in which tirofiban was used as a rescue remedy after failed thrombectomy [14]. The second is a 68-patient cohort from a 2018 study conducted by Kang et al. in which IA infusion of tirofiban was performed as a primary rescue approach in all patients after failed thrombectomy [15]. In comparison to the series reported by Zhao et al. and Kang et al, our median time to procedure was similar to Zhao et. al. (288.5 vs 292 minutes) and longer than that of Kang et al. (288.5 vs 240 minutes). Our median pre-intervention NIHSS of 11 matched that of Kang et al.; however, it was less than that of Zhao et al. (24).

**Table 3:** Comparison of our results to previous studies n, sample size; IV rt-PA, intravenous recombinant tissue plasminogen activator; MCA, middle cerebral artery; ICA, internal carotid artery; BA, basilar artery; IQR, interquartile range; NIHSS, National Institute of Health Stroke Scale; m-TICI, modified thrombolysis in cerebral infarction score; mRS, modified Rankin scale *One three-month follow-up mRS score was unavailable, so five out of the six patients were included.

				Site of Occlusion, n (%)						
Study (sample size)	Median age, years (IQR)	Males, n (%)	IV rt-PA, n (%)	MCA	ICA	BA	Median time to procedure (min), (IQR)	Median pre-intervention NIHSS (IQR)	m-TICI 2b or 3, n (%)	mRS score ≤ 2 at 3 months, n (%)
Present study (5)	64 (54–71.8)	3 (50)	3 (50)	6 (100)	0	0	288.5 (215.3–361)	11 (6–12)	6 (100)	4 (80.0)*
Kang et al., 2018 (68)	66 (57–73.8)	45 (62.5)	28 (38.9)	41 (56.9)	13 (18.1)	18 (25)	240 (168.8–357.5)	11 (6–12)	69 (95.8)	41 (56.9)
Zhao et al., 2017 (7)	78 (58.5–79.5)	5 (71.4)	2 (28.6)	6 (85.7)	0	1 (14.3)	292 (197.5–332.5)	24 (17–25)	5 (71.4)	5 (71.4)

After the failure of mechanical thrombectomy, rescue intracranial stenting is another means to achieve recanalization in patients with suspected underlying ICAS. One study found that after failed mechanical thrombectomy, rescue intracranial stenting resulted in successful recanalization in 64.6% of patients and the rescue stenting group had a significantly higher rate of good outcomes without increasing symptomatic intracranial hemorrhage or mortality [22]. The study also found that patients who had recanalization success with rescue stenting showed a good outcome rate comparable to that of the successful mechanical thrombectomy group. Though rescue stenting has good therapeutic potential, the authors of the study emphasized that rescue stenting requires acute GPI or oral antiplatelet medication to prevent in-stent thrombosis [22]. The results of our study show that even the sole use of GPI without rescue stenting can effectively treat symptomatic ICAS in selected patients by resolving acute platelet aggregation and allowing plaques to stabilize. The sole GPI treatment method treats the acute component of the stenosis, prevents its recurrence, avoids subjecting critically ill patients to the additional procedural risk of intracranial stenting, and allows for GPI titration if an intracerebral hemorrhage is found post-procedure. Our study corroborates the results from two recent studies by Zhao et al. and Kang et al. but distinguishes itself by including cases in which GPI is given as the first-line therapy in some cases identified as ICAS rather than as a rescue strategy after failed thrombectomy [14-15]. This technique may help reduce downstream embolization in these properly selected patients. Our results in this small cohort, along with the two previously published studies on this strategy, suggest that GPIs are a safe and effective revascularization strategy for AIS LVO patients with suspected underlying ICAS. Our successful results in combination with existing literature suggest that patients may benefit from greater practitioner awareness of the history and imaging factors that are suggestive of underlying ICAS in LVO patients and of this treatment option for symptomatic ICAS patients. Furthermore, as most studies on this topic have been from the continent of Asia, we provide the first experience from the Americas with the hopes of raising practitioner awareness and providing the ability to identify and successfully revascularize acute stroke LVO patients who have underlying ICAS.

The limitations of this study are its single-center, retrospective design, and the small number of patients in a case series. Future investigation is warranted to study the use of IA GPI administration for recalcitrant vessel occlusions suspected of having underlying ICAS or as a first-line therapy compared with acute intracranial stenting.

## Conclusions

We report our favorable experience using GPIs such as eptifibatide and tirofiban to restore physiologic flow in acute stroke LVO syndrome patients who were intra-procedurally determined to have underlying symptomatic ICAS. Further, we summarize the clinical and imaging characteristics that may aid in the identification of acute stroke patients presenting as LVOs who have underlying ICAS. Intra-arterial GPI infusion may be a treatment option for symptomatic large vessel occlusions or near occlusions in the appropriate clinical setting.
